# Activation of Akt by the Bacterial Inositol Phosphatase, SopB, is Wortmannin Insensitive

**DOI:** 10.1371/journal.pone.0022260

**Published:** 2011-07-14

**Authors:** Kendal G. Cooper, Seth Winfree, Preeti Malik-Kale, Carrie Jolly, Robin Ireland, Leigh A. Knodler, Olivia Steele-Mortimer

**Affiliations:** Laboratory of Intracellular Parasites, National Institutes of Allergy and Infectious Disease, National Institutes of Health, Rocky Mountain Laboratories, Hamilton, Montana, United States of America; University of Louisville, United States of America

## Abstract

*Salmonella enterica* uses effector proteins translocated by a Type III Secretion System to invade epithelial cells. One of the invasion-associated effectors, SopB, is an inositol phosphatase that mediates sustained activation of the pro-survival kinase Akt in infected cells. Canonical activation of Akt involves membrane translocation and phosphorylation and is dependent on phosphatidyl inositide 3 kinase (PI3K). Here we have investigated these two distinct processes in *Salmonella* infected HeLa cells. Firstly, we found that SopB-dependent membrane translocation and phosphorylation of Akt are insensitive to the PI3K inhibitor wortmannin. Similarly, depletion of the PI3K regulatory subunits p85α and p85ß by RNAi had no inhibitory effect on SopB-dependent Akt phosphorylation. Nevertheless, SopB-dependent phosphorylation does depend on the Akt kinases, PDK1 and rictor-mTOR. Membrane translocation assays revealed a dependence on SopB for Akt recruitment to *Salmonella* ruffles and suggest that this is mediated by phosphoinositide (3,4) P_2_ rather than phosphoinositide (3,4,5) P_3_. Altogether these data demonstrate that *Salmonella* activates Akt via a wortmannin insensitive mechanism that is likely a class I PI3K-independent process that incorporates some essential elements of the canonical pathway.

## Introduction

*Salmonella enterica* is a facultative intracellular pathogen that causes a number of diseases ranging from self-limiting gastroenteritis to systemic typhoid fever. Like many other Gram-negative pathogens, *Salmonella* use Type III Secretion Systems (T3SS) to deliver bacterial effector proteins into host cells. T3SS1, also known as the invasion associated T3SS, mediates efficient invasion of non-phagocytic eukaryotic cells, such as enterocytes in the intestinal epithelium. The invasion process has been extensively studied using cultured epithelial cells and *S. enterica* serovar Typhimurium (*S.* Typhimurium). It is characterized by the formation of localized membrane ruffles, which involves the co-operative activity of the T3SS1 effectors: SopE, SopE2 and SopB [1]. These effectors act in concert to activate the Rho family GTPases, Cdc42 and Rac, either directly, by acting as GTPase exchange factors (SopE and SopE2), or indirectly, by the generation of phosphoinositides on the cytosolic face of the plasma membrane (SopB).

In addition to its role in invasion, SopB has a number of other roles in establishing the intracellular niche [2], [3], [4], [5], [6], [7]. One of the major targets of SopB in mammalian cells is the prosurvival kinase Akt (also known as PKB) [5], [6], a serine/threonine kinase that plays central roles in a variety of cellular functions. Other bacterial pathogens also target Akt in epithelial cells, suggesting that manipulation of this kinase may be an important step in establishing infection [8], [9], [10], [11], [12], [13]. Canonical Akt activation, as illustrated by growth factor stimulation of epithelial cells, involves two sequential steps: (1) Class I PI3K-dependent membrane-translocation, followed by; (2) phosphorylation at Thr308 and Ser473, that occurs in the cell membrane [14]. The PH domain of AKT binds with high affinity to the 3′-phosphorylated lipid products of PI3K, PtdIns(3,4,5)P_3_ and PtdIns(3,4)P_2_ [15], [16], [17]. Once at the membrane, Akt is phosphorylated on Thr308 by the serine-threonine kinases PDK1 (phosphoinositide-dependent kinase 1) [18] and subsequently on Ser473 by mTORC2 (mammalian target of rapamycin complex 2) [19]. Akt phosphorylation is typically short-lived due largely to the rapid hydrolysis of PtdIns (3,4,5)P_3_ and PtdIns(3,4)P_2_ by a number of phosphoinositide phosphatases including PTEN, inositol polyphosphate 5-phosphatases and inositol polyphosphate 4-phosphatase [20], [21], [22], [23].

The mechanism of activation of Akt by SopB is not well understood. Both SopB and IpgD, a homolog from *Shigella flexneri* [24], are phosphoinositide phosphatases with homology to mammalian inositol 4-phosphatases as well as the inositol 5-phosphatase synaptojanin [25]. Phosphoinositide phosphatase activity is essential for Akt activation by either effector [5], [10], however, the mechanism by which this intersects with the canonical PI3K/Akt pathway to induce Akt activation remains unclear. Inhibition of SopB/IpgD-dependent Akt phosphorylation by the PI3K inhibitor LY294002 supports a role for PI3K [5], [10], however, a different study found that this inhibitor did not inhibit *Salmonella*-dependent accumulation of either PtdIns (3,4,5)P_3_ or PtdIns(3,4)P_2_ in membrane ruffles [26]. IpgD has been shown to activate the PI 3-kinase/Akt pathway activation via a process that involves conversion of PI(4,5)P2 into PtdIns(5)P [10], [24]. And more recently it was shown that PtdIns(5)P may act indirectly to increase Akt phosphorylation by inhibiting the PP2A phosphatase [27]. In vitro phosphatase assays have shown that SopB and IpgD have relatively low specific activity compared to mammalian homologues and that they have slightly different substrate specificities. Sop Bhas a preference for PtdIns(3,4,5)P_3_ and PtdIns(3,4)P_2_, whereas IpgD has a preference for PtdIns(4,5)P_2_ followed by PtdIns(3,4,5)P_3_ [24], [28]. Thus the involvement of PI3K in *Salmonella*-dependent Akt activation has not been definitively established.

SopB-dependent Akt activation in epithelial cells has important implications for *Salmonella* pathogenesis, particularly during the gastrointestinal phase of infection where the intestinal epithelium is targeted. To gain a better understanding of how *Salmonella* activates this critical cellular kinase in epithelial cells, we have investigated the role of PI3K, and other known components of the PI3K/Akt pathway, in SopB-dependent Akt phosphorylation and membrane localization in *Salmonella*-induced membrane ruffles.

## Results

### SopB is sufficient for Akt phosphorylation

**Figure 1:**
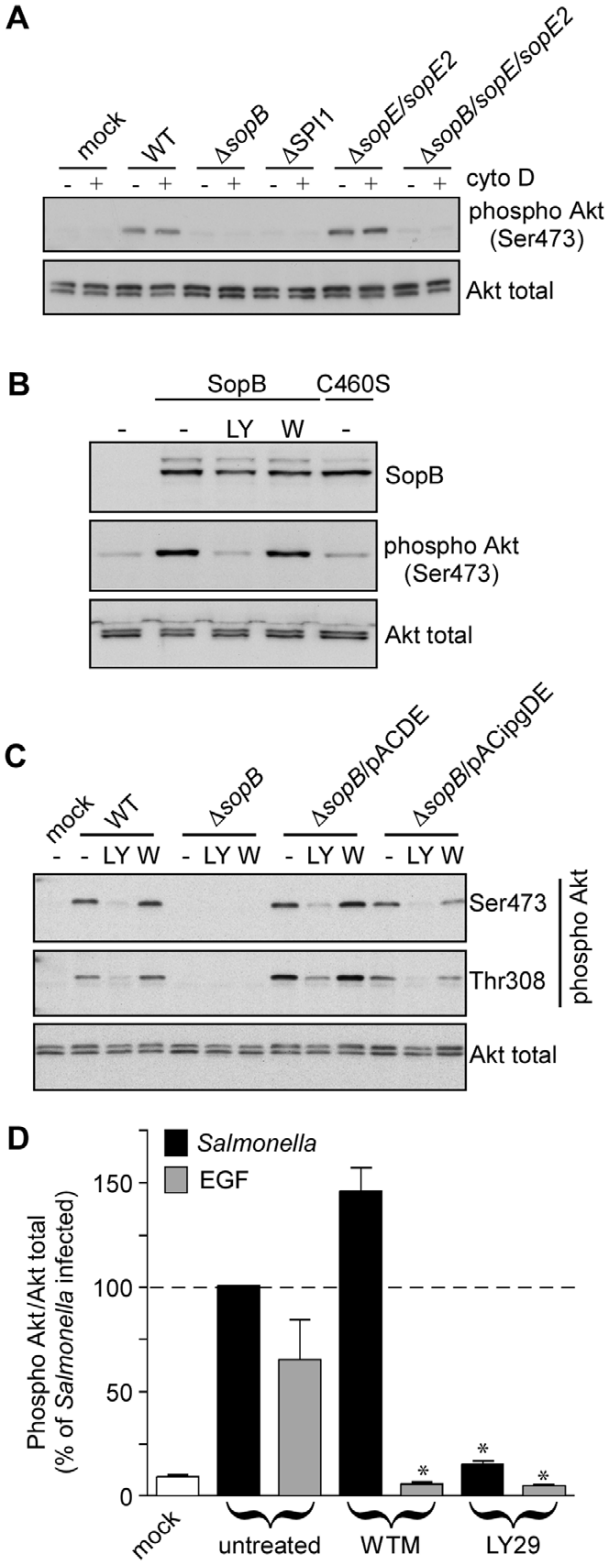
SopB–dependent Akt phosphorylation in epithelial cells is wortmannin insensitive. (A) HeLa cells were infected with *Salmonella*, either WT or the indicated mutants, for 20 min. Monolayers were then solubilized in sample buffer and processed for immunoblotting using rabbit polyclonal antibodies to detect phospho Akt (Ser473) and total Akt. Cytochalasin D (cyto D: 1 µg/ml) treated cells were incubated with drug prior to and throughout the infection. (B) HeLa cells were transfected with plasmid expressing 6His-SopB or 6His-SopB C460S for 18 hr. Monolayers were solubilized and processed for immunoblotting using antibodies against SopB, phospho Akt (Ser473) and total Akt. Where indicated, LY294002 (LY: 50 µM) or wortmannin (W:100 nM) were added for 40 min prior to sample collection. (C & D) HeLa cells were treated with EGF (50 ng/ml) for 2 min or infected with *Salmonella* for 30 min then solubilized and processed for immunoblotting (C) or ELISA (D). To compare the activities of SopB and IpgD the Δ*sopB* strain was complemented with plasmids pACDE or pACipgDE, respectively. Where indicated cells were pretreated with either LY294002 (LY29: 50 µM) or wortmannin (WTM: 100 nM) for 30 min preceding infection and inhibitor was maintained in the media for all subsequent incubations. Immunoblots are representative from three independent experiments. ELISA data represent means ± SD from three independent experiments (* *P*<0.05, significantly different from untreated).

Several features of *Salmonella* pathogenesis require the concerted actions of multiple T3SS1 effectors. In particular, SopB cooperates with SopE and SopE2 to induce the actin rearrangements leading to invasion [29]. To investigate whether these, or other effectors, contribute to SopB-dependent *Salmonella*-mediated Akt phosphorylation, HeLa cells were infected with mutant *S.* Typhimurium strains that lacked either specific effectors or the ability to translocate them. Akt phosphorylation was then assessed by immunoblotting using phospho-specific antibodies that recognize Akt when it is phosphorylated at Ser473 or Thr308 (Figure 1A). As shown previously, wild type (WT) *Salmonella* induces Akt phosphorylation whereas a *sopB* deletion mutant, Δ*sopB*, does not [5]. A strain lacking SopE and SopE2 (Δ*sopE*/*sopE2*) induced Akt phosphorylation levels comparable to WT, whereas the triple mutant Δ*sopE*/*sopE2/sopB* was indistinguishable from the Δ*sopB* strain. A ΔSPI1 mutant, which lacks the T3SS1 structural and regulatory components and is unable to translocate any T3SS1 effectors into host cells, also did not induce Akt activation. Since several of these mutants are invasion defective, we confirmed that invasion *per se* is not required for Akt activation by pretreating cells with cytochalasin D to disrupt the actin cytoskeleton. Cytochalasin D inhibits bacterial invasion (not shown and [30]) but had no effect on the ability of WT *Salmonella* to induce Akt phosphorylation in HeLa cells (Figure 1A), confirming that effector translocation, but not bacterial invasion, is required for *Salmonella*-induced Akt phosphorylation. To rule out a requirement for any other bacterial factors, His-tagged SopB (6His-SopB) was expressed from a mammalian expression plasmid in HeLa cells. Akt phosphorylation was increased in cells expressing 6His-SopB compared to control cells (no plasmid) or cells expressing the catalytically inactive SopB C460S mutant [5](Figure 1B). Together these experiments show that SopB phosphatase activity is the only bacterial factor required for *Salmonella*-mediated Akt phosphorylation in HeLa cells.

### SopB-dependent Akt activation is wortmannin-insensitive

**Figure 2:**
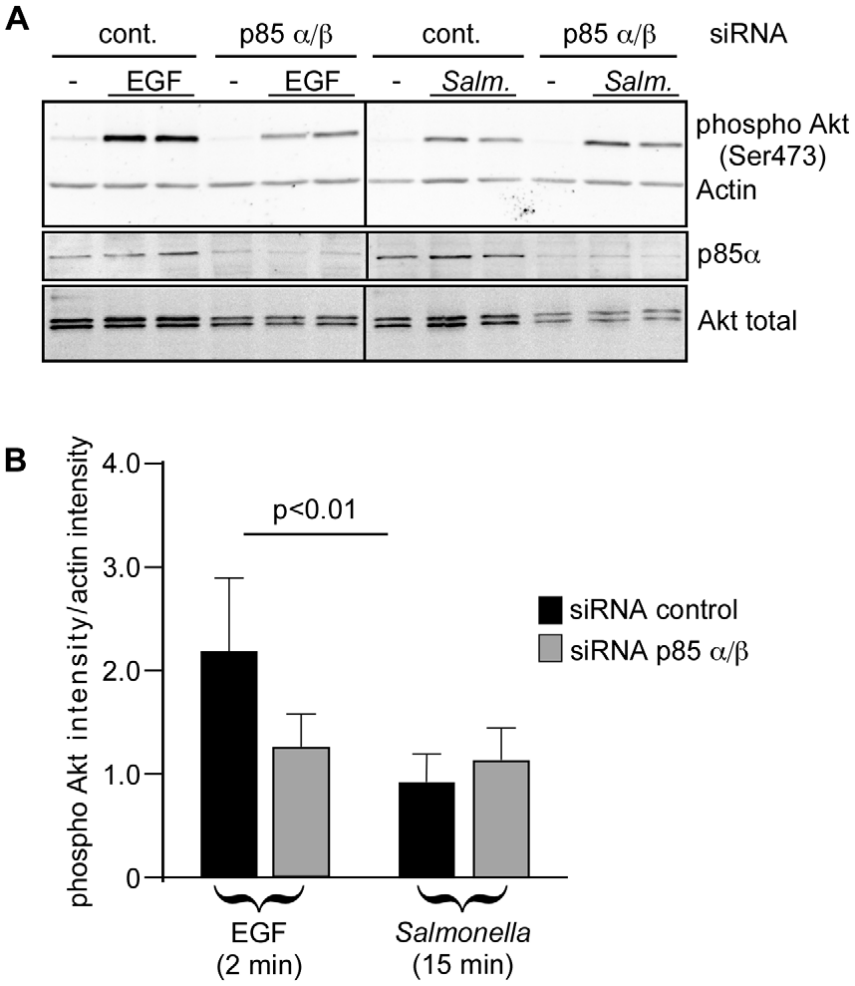
The class I PI3K regulatory subunits p85α and p85ß are not required for SopB-mediated Akt phosphorylation. HeLa cells were transfected with siRNAs, specific for p85α and p85ß, for 72 hr then either treated with EGF or infected with *Salmonella* WT. For siRNA control siRNA specific for AKT3 was used (cont.). Monolayers were then solubilized in sample buffer and processed for immunoblotting using antibodies to detect phospho Akt (Ser473), total Akt or p85α. (A) Representative immunoblot showing p85α knockdown efficiacy and effect on Akt phosphorylation in infected or EGF treated cells. (B) Quantification of Akt phosphorylation estimated by densitometry. Shown are the means ± SD from three independent experiments.

**Figure 3:**
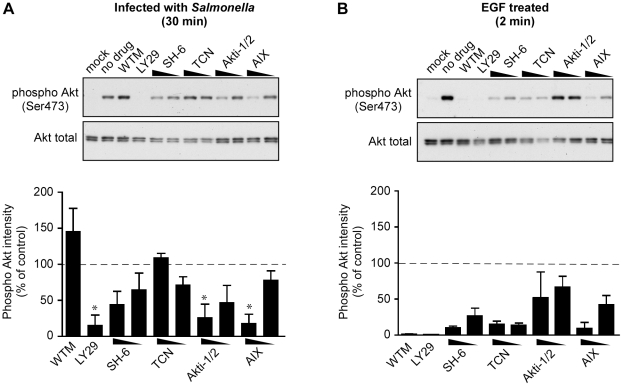
PI3K/Akt inhibitors differentially affect *Salmonella*-induced and EGF-induced Akt phosphorylation in epithelial cells. HeLa cells were pretreated with PI3K/Akt inhibitors for 30 min and then infected with *Salmonella* for 30 min (A) or treated with EGF (50 ng/ml) for 2 min (B). Monolayers were then solubilized in sample buffer and processed for immunoblotting using antibodies to detect phospho Akt (Ser473) and total Akt. Inhibitors used were; Wortmanin (WTM:100 nM), LY294002 (LY29: 50 µM), SH-6 (20 µM, 10 µM), TCN (20 µM, 10 µM), Akti-1/2 (0.1 µM, 0.05 µM) and AIX (10 µM, 5 µM). The graphs below each panel show the quantification of Akt phosphorylation estimated by densitometry. Shown are the means ± SD from three independent experiments (* *P*<0.05, significantly different from untreated).

We next investigated the role of PI3K in SopB-induced Akt phosphorylation using the PI3K inhibitors wortmannin and LY294002. HeLa cells expressing 6His-Sop Bwere treated with the inhibitors and Akt phosphorylation assessed by immunoblotting (Figure 1B). Surprisingly, wortmannin had no effect on SopB-dependent Akt phosphorylation in this system. In contrast, LY294002 completely inhibited SopB-dependent Akt phosphorylation. To confirm that this was not an artifact of ectopic expression we next compared the inhibitory activities of LY294002 and wortmannin in HeLa cells infected with *Salmonella*. Cells were pretreated with inhibitors for 30 min then infected with *Salmonella* for 30 min in the presence of the inhibitors. Subsequently we assessed the levels of phosphorylated Akt either by immunoblotting or ELISA (Figure 1C and D). In agreement with the results obtained with ectopically expressed SopB, SopB-dependent Akt phosphorylation in *Salmonella*-infected cells was efficiently inhibited by LY294002 but not by wortmannin. In these experiments, and subsequently (Figure 2 and 3), EGF stimulation of HeLa cells was used as a positive control for activation of the canonical PI3K/Akt pathway. Both of the PI3K inhibitors completely inhibited EGF-dependent Akt phosphorylation (Figure 1D). Control experiments were also carried out in which wortmannin was added to cells for 30 min or 3 hr prior to infection with *Salmonella* or EGF treatment. Irrespective of the pre-incubation period, wortmannin efficiently inhibited Akt phosphorylation in HeLa cells stimulated with EGF but not in cells infected with *Salmonella* (Figure S1). These experiments were repeated in human (FHs 74 Int) and rat (IEC-18) intestinal epithelial cells that are physiologically relevant for *Salmonella* pathogenesis (data not shown). In these cell lines *Salmonella*-induced Akt phosphorylation was also insensitive to wortmannin, thus wortmannin-insensitivity seems to be a characteristic of this pathway in epithelial cells.

The Akt phosphorylation defect of Δ*sopB Salmonella* can be rescued by plasmid expressed SopB or the *Shigella* homologue IpgD [25]. Using the plasmids pACDE, which encodes both SopB and its chaperone SigE, and pACipgDE, which encodes IpgD and its chaperone IpgDE, we directly compared SopB- and IpgD-dependent Akt phosphorylation in infected HeLa cells. In both plasmids, expression is under the transcriptional control of the *sopB* promoter [25]. Like SopB, IpgD efficiently induced Akt phosphorylation, which was inhibited by LY294002 but not wortmannin (Figure 1C). Thus SopB and IpgD induce Akt phosphorylation via a similar wortmannin-insensitive mechanism.

Since the differential sensitivity to the pharmacological inhibitors wortmannin and LY294002 was both unexpected and difficult to interpret, we next sought to verify whether or not class I PI3K is required for *Salmonella*-induced Akt activation. To do this we used RNAi-mediated knockdown to deplete the p85α and p85ß regulatory subunits of class I PI3K. Cells were transfected with siRNA 48 hr prior to infection with *Salmonella* for 15 min. As shown in Figure 2, depletion of p85 resulted in significant inhibition of EGF-induced Akt-phosphorylation but had no effect on *Salmonella*-induced Akt-phosphorylation. Furthermore, a time course experiment showed no requirement for PI3K in *Salmonella*-induced Akt-phosphorylation up to 3 hr post-infection (Figure S2). Together the above experiments indicate that the *Salmonella*-induced phosphorylation of Akt is not dependent on class I PI3K.

### Differential effects of Akt inhibitors on SopB- and EGF-induced phosphorylation of Akt

Having shown a difference between *Salmonella*-mediated and EGF-mediated Akt activation using the PI3K inhibitor wortmannin, we next targeted post-PI3K steps in the Akt-activation pathway using a panel of pharmacological inhibitors. These included: SH-6, a phosphatidylinositol analog that prevents phosphorylation of Akt [31], [32]; Triciribine (TCN), a cell-permeable tricyclic nucleoside that selectively inhibits the cellular phosphorylation/activation of Akt without affecting either PI3K or PDK [33]; Akti-1/2, a PH domain dependent allosteric inhibitor that preferentially inhibits Akt1 and Akt2 [34]; and Akt inhibitor×[10-(4′-(N-diethylamino)butyl)-2-chlorophenoxazine, HCl] (AIX), a PH domain independent inhibitor of Akt kinase activity [35]. HeLa cells were treated with Akt inhibitors for 30 min then either infected with *Salmonella* for 30 min or treated with EGF for 2 min. AIX was the only one of these inhibitors that inhibited *Salmonella*- and EGF-stimulated Akt phosphorylation with similar efficiency (compare Figure 3A and B). Two of the inhibitors, SH-6 and TCN, had no significant effect on *Salmonella*-induced Akt phosphorylation when used at concentrations that caused inhibition of EGF-stimulated Akt phosphorylation. In contrast, Akti-1/2 had no effect on EGF-stimulated Akt phosphorylation at the concentrations used here (0.05 µM–0.1 µM) but did significantly reduce *Salmonella*-induced Akt phosphorylation at 0.1 µM. Altogether, these results confirm our initial findings with the PI3K inhibitor wortmannin; that SopB-dependent Akt phosphorylation is occurring via a mechanism distinct from the canonical PI3K/Akt pathway.

### Rictor and PDK1 are involved in SopB-dependent Akt phosphorylation

**Figure 4:**
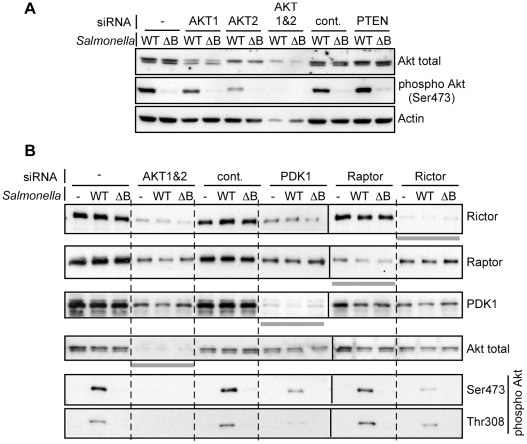
Both PDK1 and rictor are required for *Salmonella*-induced Akt phosphorylation. A and B. HeLa cells were transfected with the indicated SMART pool siRNA for 48 hr then infected with *Salmonella* WT or Δ*sopB*. For siRNA control SMART pool siRNA specific for AKT3 was used (cont.). After 30 min, monolayers were solubilized and processed for immunoblotting. Antibodies were used to detect phospho-Akt (Ser473 or Thr308), total Akt, PDK1, raptor, rictor or actin. Gray bars underneath the individual panels highlight the efficiency of each siRNA knockdown.

To verify the above data and also determine the requirement for other known components of the PI3K/Akt pathway in SopB-mediated Akt phosphoylation, we used RNAi-mediated knockdown to deplete proteins directly involved in Akt regulation (Figure 4). First, we performed targeted knockdown using isoform-specific siRNAs to compare the roles of Akt1 and Akt2, the two Akt isoforms present in HeLa cells. Cells were transfected with siRNA 48 hr prior to infection with *Salmonella* for 30 min. The levels of total Akt (Akt1 and Akt2), phospho Akt (Akt1 and Akt2) and actin were then assessed by immunoblotting. In HeLa cells the pan Akt antibody that we used to detect total Akt, recognizes both Akt1 (upper band) and Akt2 (lower band). Knockdown efficacy was better for Akt2 than Akt1. Negative control siRNA targeting Akt3, an isoform not expressed in HeLa cells, did not affect Akt1 and Akt2 levels and had no effect on *Salmonella*-dependent Akt phosphorylation. Depletion of either Akt1 or Akt2 resulted in reduced levels of Akt phosphorylation although Akt2 depletion had a more pronounced effect (Figure 4A). Depletion of both Akt1 and Akt2 caused almost complete abrogation of Akt phosphorylation as previously shown [6], but also caused loss of cell growth and/or viability as in dicated by the decrease in actin. These data show that *Salmonella* can induce phosphorylation of both Akt1 and Akt2 in infected HeLa cells.

Down-regulation of growth factor mediated Akt phosphorylation is dependent on phosphatase and tensin homologue deleted on chromosome 10 (PTEN) which dephosphoylates PtdIns(3,4,5)P_3_. However, targeted knockdown of PTEN with siRNA had no apparent effect on the amount of Akt phosphorylation in HeLa cells infected with *Salmonella* for 30 min (Figure 4A) or in extended (3 hr) time-course experiments (data not shown).

Phosphorylation of Akt at Thr308 and Ser473 is mediated by the Akt kinases, PDK1 and mTORC2 respectively [19], [36]. We assessed the role of these kinases using siRNA targeting PDK1 or Rictor, the defining component of the multisubunit complex mTORC2. In cells depleted of PDK1 and then infected with WT *Salmonella* for 30 min, we observed a strong reduction in Thr308 phosphorylation as well as a detectable reduction in Ser473 phosphorylation (Figure 4B). In contrast, in mTORC2 depleted cells Ser473 phosphorylation was preferentially reduced. As an additional control, we also depleted raptor, which is complexed with mTOR in mTORC1, but this had no effect on Akt phosphorylation. Collectively, these data demonstrate a requirement for both PDK1 and mTORC2 in the *Salmonella*-induced activation of Akt.

### PDK1 and rictor, are recruited to *Salmonella*-induced ruffles independent of SopB

**Figure 5:**
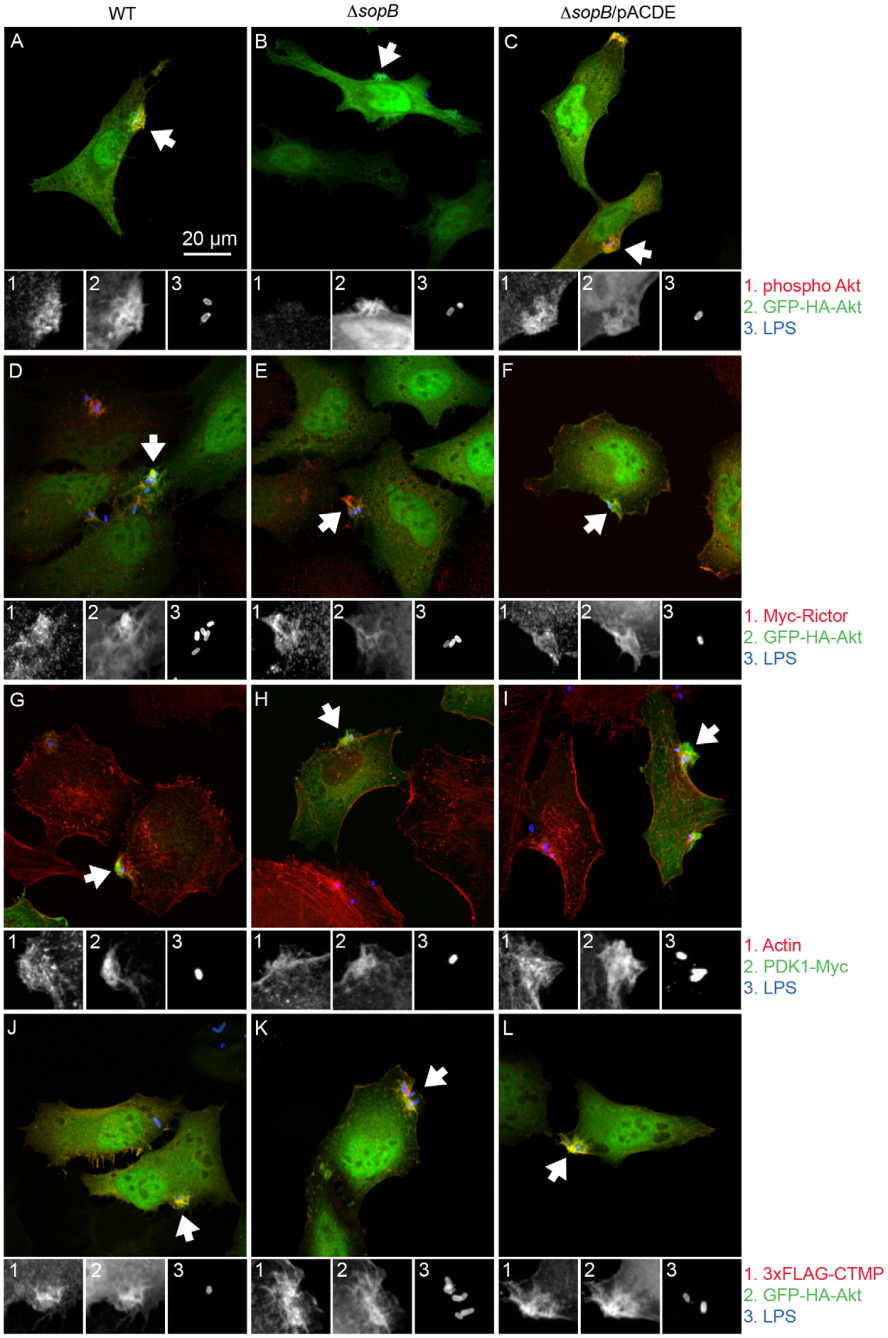
Akt regulators accumulate in *Salmonella* induced ruffles. HeLa cells were transfected with plasmids encoding epitope-tagged proteins as indicated. After 20 hr they were infected with *Salmonella* for 30 min, then fixed and processed for immunofluorescence microscopy. GFP-HA-Akt transfected cells were stained for *Salmonella* LPS (Cy5) and phospho Akt Ser473 (AF568: A–C), Myc (AF568: D–F) or 3×FLAG (AF568: J–L). Cells expressing PDK1-Myc (G–I) were stained for LPS (Cy5), Myc (AF488) and actin filaments (phalloidin-AF568) to reveal the ruffles.

Having shown that *Salmonella*-induced phosphorylation of Akt is dependent on PDK1 and rictor we next sought to confirm that these kinases are translocated to the plasma membrane during infection. The dominant characteristic of *Salmonella* invasion of epithelial cells is the formation of membrane ruffles and Akt is specifically translocated to the ruffle where it is phosphorylated [6]. To determine whether the Akt kinases are also translocated to the ruffles we used transiently expressed myc-tagged PDK1 and rictor fusion proteins since the endogenous proteins were below the levels of detection in our system (not shown). As shown in Figure 5 both PDK1-Myc and Myc-rictor were recruited to ruffles induced by WT *Salmonella*.

**Figure 6:**
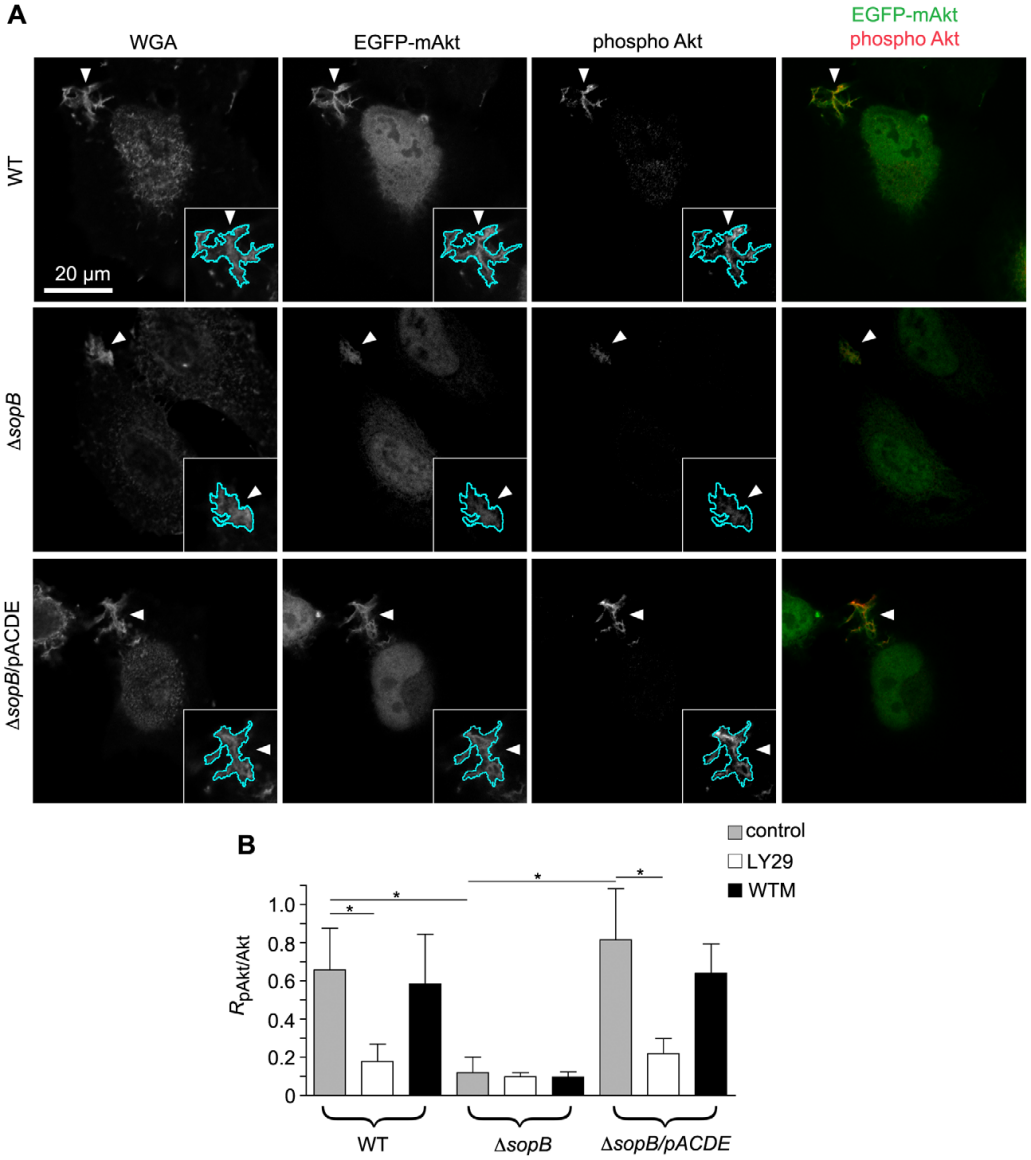
Accumulation of phospho Akt in *Salmonella*-induced ruffles is wortmannin insensitive. HeLa cells expressing EGFP-mAkt were infected with *Salmonella* for 20 min then fixed and processed for immunofluorescence microscopy. Cells were doubly stained for plasma membrane (Cy5-WGA) and phospho Akt Ser473 (AF568). (A) Representative images with ruffles outlined to show phospho Akt in ruffles induced by WT *Salmonella*. In comparison, phospho Akt levels are much lower in ruffles induced by the Δ*sopB* mutant, unless the mutant is complemented *in trans* with *sopB* (pACDE). (B) Semi-quantitative analysis of phospho Akt (Ser473) levels (*R*_pAkt/Akt_) in membrane ruffles. Where indicated, cells were pretreated with wortmannin (WTM: 100 nM) or LY294002 (LY29: 50 µM) for 30 min prior to infection and maintained throughout. Data are the means ± SD from three independent experiments (* *P*<0.05).

Intriguingly, although SopB is required for *Salmonella* induced phosphorylation of Akt, no requirement has been demonstrated for SopB in membrane translocation. On the contrary, Akt is apparently enriched in ruffles induced by Δ*sopB Salmonella* (Figure 5 and [6]). Here we found that PDK1 and rictor are also translocated to ruffles induced by the Δ*sopB* strain (Figure 5). These experiments indicate that Akt, PDK1 and rictor are translocated to *Salmonella*-induced ruffles independent of SopB activity. This does not explain why Akt phosphorylation is strictly SopB dependent. One possibility is that a negative regulator of Akt phosphorylation could be involved in the absence of SopB. We analyzed the localization of CTMP, a 27-kDa protein that has been shown to regulate the activity of Akt by associating with it at the plasma membrane [37], [38]. However, in HeLa cells co-expressing FLAG-CTMP and GFP-Akt, CTMP co-localized with Akt in ruffles induced by either WT *Salmonella* or the Δ*sopB* mutant. Altogether these experiments did not reveal any requirement for SopB in localization of Akt kinases or CTMP to plasma membrane ruffles.

### Semi-quantitative analysis of SopB-dependent Akt recruitment and phospholipid changes in *Salmonella*-induced membrane ruffles

**Figure 7:**
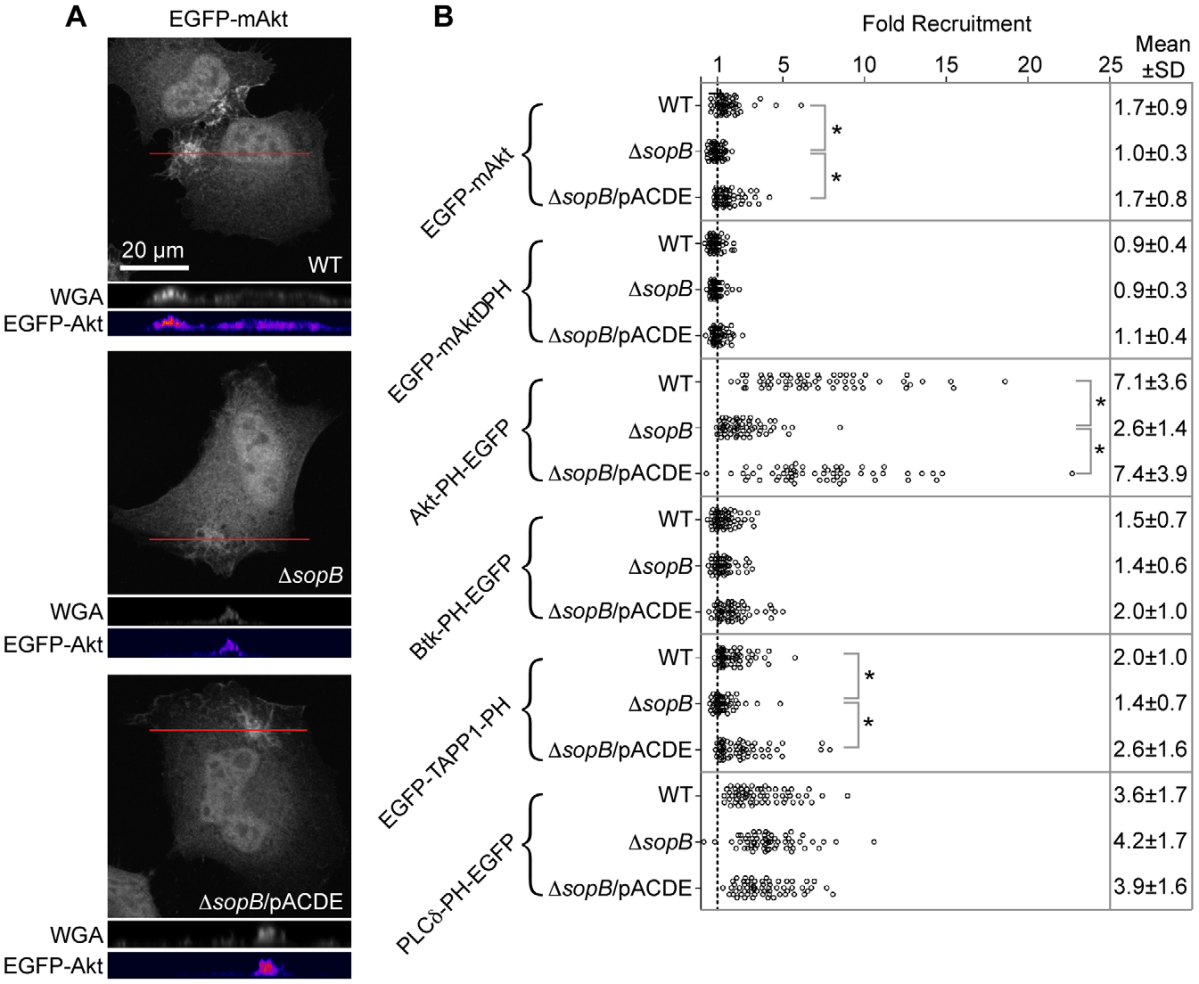
Enrichment of PH domain lipid-binding probes in *Salmonella*-induced ruffles. HeLa cells transfected with plasmids encoding EGFP-fusions to full length Akt (EGFP-mAkt) or isolated PH domains as indicated were infected with *Salmonella* for 30 min, then fixed and processed for immunofluorescence microscopy. Cells were stained for plasma membrane (Cy5-WGA) and *Salmonella* LPS (AF568). (A) Representative images to show EGFP-mAkt accumulation in ruffles induced by *Salmonella* WT, the Δ*sopB* mutant or Δ*sopB* complemented *in trans* with *sopB* (pACDE). Orthogonal sections show WGA (grayscale) and EGFP-Akt (grayscale converted to a heatmap using the “FIRE” look up table of ImageJ) corresponding to the red lines on the projections. (B) Analysis of GFP fusion enrichment in membrane ruffles. Shown is combined data from three independent experiments. Each dot represents one ruffle. P values were obtained by ANOVA and Tukey's post hoc analysis (* *P*<0.05).

Although the visual comparison of ruffles did not reveal a requirement for SopB in Akt, PDK1 or rictor recruitment, we considered that subtle changes in membrane recruitment might not be detected by this method. We therefore used a semi-quantitative microscopy-based method to obtain a more accurate measurement of Akt phosphorylation and protein recruitment in *Salmonella*-induced ruffles. This method involves comparison of the protein of interest to a plasma membrane reference marker, fluorescently conjugated wheat germ agglutinin (WGA), in order to compensate for the variable amount of membrane in ruffles [39]. Single optical sections through ruffles were then obtained using a spinning disc confocal microscope. It should be noted that *Salmonella*-induced ruffles protrude above the surface of the cell, so that the majority of these z-sections do show not the main body of the cell (Figure 6A). Compare with the images shown in Figure 7A, where YZ single sections are included to illustrate the intensity and height of ruffles compared to the rest of the cell. When the ratio of intensity of phospho Akt/total Akt (*R*_pAkt/Akt_) was calculated for individual ruffles induced by WT *Salmonella* the *R*_pAkt/Akt_ was approximately 3-fold higher than that in ruffles induced by the Δ*sopB* strain (Figure 6B). Complementation with plasmid borne SopB restored the WT phenotype. When LY294002-treated cells were infected with *Salmonella* expressing SopB the *R*_pAkt/Akt_ value was reduced to the level of that induced by the Δ*sopB* strain. In contrast, wortmannin had no effect on the *R*_pAkt/Akt_ values. Thus measurement of Akt phosphorylation in ruffles provides results strikingly similar to those obtained by immunoblotting for whole cell lysates and reiterates the finding that wortmannin does not inhibit SopB-dependent Akt phosphorylation (Figure 1).

Next we used a similar microscopy based semi-quantitative method to measure recruitment of Akt to ruffles (Figure 7). In this assay the average pixel intensity (background subtracted) in ruffles is compared to the average pixel intensity in the cytosol (background subtracted) [Recruitment = (GFP_Ruffle_−GFP_back_)/(GFP_cyto_−GFP_back_)], and any value greater than 1 indicates recruitment. This method revealed a subtle but significant requirement for SopB in Akt recruitment that was not apparent by visual assessment (Figure 7B). In ruffles induced by WT *Salmonella* recruitment was higher than in ruffles induced by the Δ*sopB* strain and complementation of the Δ*sopB* strain restored the WT phenotype (WT = 1.7±0.9; Δ*sopB* = 1.0±0.3; Δ*sopB*/pACDE = 1.7±0.8). An Akt construct, EGFP-mAktΔPH, lacking the phophoinositide binding PH domain, and therefore unable to bind membranes, was not enriched in *Salmonella*-induced ruffles (WT = 0.9±0.4; Δ*sopB* = 0.9±0.3; Δ*sopB*/pACDE = 1.1±0.4). In contrast, the PH domain of Akt (Akt-PH-EGFP) was efficiently recruited to ruffles via a SopB-dependent process (WT 7.1±3.6; Δ*sopB* 2.6±1.4; Δ*sopB*/pACDE 7.4±3.9). Translocation to the plasma membrane of the Akt PH domain can be mediated by PtdIns(3,4,5)P_3_ and/or PtdIns(3,4)P_2_ [40], [41]. To determine whether both of these phosphoinositides are enriched in in ruffles we used GFP fusions to the PH domains of Btk and TAPP1 which bind PtdIns(3,4,5)P_3_ and PtdIns(3,4)P_2_ respectively [40], [42]. Only EGFP-TAPP1-PH showed statistically significant recruitment to ruffles in a SopB-dependent manner (WT = 2.0±1.0; Δ*sopB* = 1.4±0.7; Δ*sopB*/pACDE = 2.6±1.6). This suggests that, in *Salmonella*-induced ruffles, SopB activity leads to an enrichment of PtdIns(3,4)P_2_, rather than PtdIns(3,4,5)P_3_. Finally, we analyzed recruitment of the PH domain of phospholipase C delta (PLC-δ), a probe for PtdIns(4,5)P_2_ [42], [43]. This probe (PLCδ-PH-EGFP) confirmed that PtdIns(4,5)P_2_ is enriched in *Salmonella*-induced ruffles (WT = 3.6±1.7; Δ*sopB* = 4.2±1.7; Δ*sopB*/pACDE = 3.9±1.6). Although we could not detect any statistically significant dependence on SopB, it should be stated that this technique assesses the total amount of probe in ruffles and would not reveal differences within sub-regions of the ruffles. For example, depletion of PtdIns(4,5)P_2_ at the apex of the phagocytic cup as has previously been shown [2].

## Discussion

While it has been well established that the PI3K/Akt pathway is modulated by many viruses and plays an important role in the establishment of viral infection [44], the appropriation of Akt by pathogenic bacteria is less well understood [5], [6], [10], [11], [45], [46], [47]. *Salmonella*, and other intracellular bacteria [9], use Akt activation to block or delay apoptosis in infected cells. Given the diverse cellular roles of Akt, it is likely to have additional functions during bacterial infection.

In this study, we first showed that the *Salmonella* effector protein SopB is necessary and sufficient for Akt phosphorylation in HeLa cells. To gain a better understanding of the role of Akt in *Salmonella* pathogenesis we then compared SopB-mediated Akt activation with the canonical EGF signaling pathway common to all epithelial cells. Using different approaches we assessed the two essential steps in Akt activation i.e. membrane translocation and phosphorylation. The most striking difference that our study revealed is that the irreversible PI3K inhibitor wortmannin is unable to inhibit either of these steps in *Salmonella*-infected HeLa cells. An obvious interpretation of this is that SopB-dependent Akt activation is independent of class I PI3K, supported by the finding that depletion of the p85 regulatory subunit of class I PI3K had no effect on this pathway. Surprisingly, the more specific PI3K inhibitor LY294002 did inhibit both membrane translocation and phosphorylation of Akt in *Salmonella* infected cells. However, LY294002 does have other intracellular targets [48], including: casein kinase-2, GSK3α and GSK3ß, as well as p97/VCP, a member of the type II AAA ATPase family [49]. Several other potential targets, DNA-PK, PI4K and mTOR, can be excluded since they are equally sensitive to wortmannin [50], [51], [52]. We also found that SopB-dependent Akt phosphorylation was less sensitive than EGF-induced phosphorylation to two small molecule inhibitors of AKT. SH-6 is a phosphatidylinositol analog that competes with PI3K for PtdIns(4,5)P_2_ [32] whereas TCN is a cell-permeable tricyclic nucleoside that inhibits Akt phosphorylation [33]. One possibility is that the SopB pathway engages a mammalian PI3K other than the canonical class I PI3K, although this is unlikely since WTM does not show significant isoform specificity. A final alternative is PI3K-independent activation of Akt. This is not without precedent since both cAMP/protein kinase A and dopamine have been shown to elicit wortmannin-insensitive Akt activation [53], [54], [55], [56]. Despite the above differences between the SopB-mediated and EGF-mediated pathways of Akt activation our data suggest that the Akt kinases, PDK1 and mTORC2, are essential components in both cases.

To get a better understanding of the role of SopB in recruitment of signaling components we also investigated recruitment of proteins and phosphoinoside specific PH domains to membrane ruffles. This semi-quantitative method revealed that Akt enrichment is SopB dependent, whereas in a previous study where enrichment was simply assessed visually, we could not detect any requirement for SopB [5]. Furthermore, the PH domain translocation experiments indicated that SopB induces a localized increase in PtdIns(3,4)P_2_ rather than PtdIns(3,4,5)P_2_ in *Salmonella*-induced ruffles. This suggests that Akt phosphorylation in the *Salmonella*-induced ruffle is dependent on PtdIns(3,4)P_2_ rather than PtdIns(3,4,5)P_2_. Further studies are required to determine the roles of these phosphoinositides in SopB-dependent Akt activation. Interestingly, studies on the *S. flexneri* effector protein IpgD, a homolog of SopB, have shown that sustained Akt phosphorylation is mediated by IpgD-dependent generation of PtdIns(5)P [10] and indeed SopB causes localized conversion of PI(4,5)P_2_ to PI(5)P in regions of *Salmonella*-induced plasma membrane ruffles [2], [57]. One possible effect of increased PtdIns(5)P is to prevent the dephosphorylation of Akt by inhibiting the catalytic subunit of PP2A phosphatases [27]. However, these studies also found that PI3K played an essential role in IpgD-dependent Akt-phosphorylation. Unfortunately, PtdIns(5)P is a rare phosphoinositide [48], making it very difficult to detect and it remains poorly understood.

In conclusion, we have shown that *Salmonella* induces Akt activation via a wortmannin insensitive mechanism that probably involves a novel class I PI3K-independent pathway. Why *Salmonella* have not simply tuned into the canonical pathway is unclear, but one possibility is that it could allow the targeting of different downstream proteins. The molecular mechanisms involved in this process remain unknown, however, the work presented here provides a foundation for future experiments that should lead to a better understanding of bacterial pathogenesis as well as the multi-faceted essential kinase Akt.

## Materials and Methods

### Materials

Primary antibodies, mouse monoclonal antibodies to Myc and horseradish peroxidase (HRP)-conjugated secondary antibodies were obtained from Cell Signaling Technology unless otherwise noted. Fluorescently labeled Alexa Fluor (AF)-conjugated antibodies and phalloidin were from Invitrogen. Cy5-conjugated antibodies were from Jackson ImmunoResearch. Rabbit polyclonal antibodies to *Salmonella* LPS (Salmonella O Antiserum Group B Factors 1, 4, 5, 12) were from BD. Anti-FLAG antibodies were from Sigma. Other chemicals were from EMD Biosciences except where indicated. Cytochalasin D was from Sigma. LY294002 was from Enzo Life Sciences.

### Cell Culture

HeLa (human cervical adenocarcinoma, ATCC CCL-2) cells were grown at 37°C in 5% CO_2_ in Eagle's minimal essential medium (EMEM) (Mediatech) supplemented with 10% (v/v) heat-inactivated fetal bovine serum (Invitrogen). Cells were passaged every three to four days and used for experiments within 15 passages of receipt from ATCC.

### Bacterial strains and plasmids

*Salmonella enterica* serovar Typhimurium SL1344 wild type and the mutants ΔSPI1::kan, Δ*sopB* and *sopE*::*aphT/sopE2*::pM218 (Tet^R^) (M202^E-E2-^) were as previously described [5], [58], [59]. The Δ*sopB/sopE*::*aphT/sopE2*::pM218 was constructed by sequential phage P22-mediated transduction of the Δ*sopE*::*aphT* and Δ*sopE2*::pM218 alleles of M202^E-E2-^ into SL1344 Δ*sopB*.

The complementing plasmids pACDE and pACipgDE have been described previously [25]. EGFP-mAkt [39], Myc-PDK1 [60], green fluorescent protein (GFP)-HA-Akt [41], enhanced GFP (EGFP)-TAPP1-PH [61], Akt-PH-EGFP, PLCδ-PH-EGFP and Btk-PH-EGFP [43] have all been described previously. Myc-Rictor [62] was purchased from Addgene (plasmid #11367). To remove the PH domain of Akt, EGFP-mAkt was used as template in inverse PCR with the oligonucleotides Akt deltaPH-129F (CCC AAG CTT TCA GGG GCT GAA GAG ATG) and Akt deltaPH-4R (CCC AAG CTT TAC GTC GTT CAT AGA TCT). The amplicon was digested with HindIII and self-ligated to create EGFP-mAktΔPH. CTMP was amplified from a Human Kidney Creator SMART cDNA library (Clontech) with the oligonucleotides hCTMP-3FLAG-Bgl5 (5′ GGA AGA TCT GCT GAG GAG CTG CGC CGC G 3′) and hCTMP-3FLAG-Sal3 (5′ A CGC GTC GAC TTA TGT CAG ACT TTT AGC AGG ATT CAG 3′). The resulting amplicon was cloned into pCR2.1 TOPO (Invitrogen), released by EcoRI digestion and ligated into EcoRI-digested p3×FLAG-CMV™-7.1 (Sigma) to create 3×FLAG-CTMP.

For ectopic expression of SopB, *sopB* was amplified from pACDE [25] or pACDE C460S [6] with the oligonucleotides 6His-SigD-F (5′ CGC GGA TTC AAA TAC AGA GCT TCT ATC AC 3′) and 6His-SigD-R (5′ CCG CTC GAG TCA AGA TGT GAT TAA TGA AGA 3′) (engineered restriction sites are underlined). The resulting amplicons were digested with BamHI and XhoI and ligated into the corresponding sites of pcDNA3.1/His A (Invitrogen) to create 6His-SopB and 6His-SopB C460S, respectively.

### Bacterial Infection of Mammalian Cells

Cells were seeded into 6-well plates (2.0×10^5^ cells/well), 10 cm tissue culture dishes (1.6×10^6^ cells/dish) or glass coverslips in 24-well plates (5.0×10^4^ cells) to yield monolayers of 75–85% confluency after 16–20 h. SPI1-induced bacteria were prepared by diluting 0.3 ml of overnight LB-Miller culture in 10 ml of fresh LB-Miller and incubating at 37°C with shaking (225 rpm). At late log phase (3.5 h), the bacteria were pelleted at 8,000× *g* for 2 min and resuspended in an equal volume of Hanks' balanced salt solution (Mediatech) or phosphate-buffered saline. This suspension of invasive bacteria was then used to inoculate HeLa cells (multiplicity of infection = 100) and invasion was allowed to proceed for 10 min at 37°C in 5% CO_2_. Following invasion, extracellular bacteria were removed by washing with HBSS and the cells were then incubated with serum-free EMEM for 20 min. For experiments requiring longer incubations, gentamicin (50 µg/ml) was added 30 min post-infection and reduced to 10 µg/ml at 90 min post-infection, to kill any extracellular bacteria. Where indicated, cells were treated with inhibitors for 30 min immediately before infection and drugs were maintained in media thereafter. For serum starvation cells were incubated in serum-free EMEM for 3–3.5 h immediately before infection and also for subsequent steps.

### Immunoblotting

Monolayers of infected HeLa cells in 6 well plates were solubilized in hot SDS-PAGE sample buffer (100 µl/well) at the indicated times and SDS-PAGE and immunoblotting were performed as described previously [4]. Rabbit polyclonal anti-Akt, rabbit monoclonal anti-total Akt (pan) (11E7) (cat # 4685), rabbit polyclonal anti-phospho-Akt Ser473, rabbit monoclonal anti-phospho-Akt (Ser473) (193H12), rabbit monoclonal anti-phospho-Akt (Thr308) (D9E), rabbit polyclonal anti-PDK1, rabbit polyclonal anti-raptor, rabbit polyclonal anti-rictor (BL2181, Bethyl Laboratories), mouse monoclonal anti-PI3K p85α (clone AB6, Millipore) or mouse monoclonal anti-actin (C-2, Santa Cruz Biotechnology) were used at a 1∶1,000 to 1∶20,000 dilution in blocking buffer [Tris buffered Saline, 0.1% (v/v) Tween 20, 1% bovine serum albumin]. Secondary antibodies, horseradish peroxidase-conjugated goat anti-rabbit or horse anti-mouse IgG, were diluted 1∶5,000 in Tris buffered saline, 0.1% (v/v) Tween 20, 5% (w/v) skim milk powder. For chemiluminescent detection the SuperSignal West Femto Substrate Kit or SuperSignal West Pico Substrate Kit were used according to the manufacturer's instructions (Thermo). Immunoblotting with rabbit polyclonal antibodies to SopB was as previously described [63], [64].

### ELISA

HeLa cells in 10 cm dishes were infected with *Salmonella* as described above. Samples were prepared and the level of Akt phosphorylation was assessed using the PathScan® Phospho-Akt1 (Ser473) Sandwich ELISA Kit (Cell Signaling Technology) according to the manufacturer's instructions.

### Transient expression of proteins in HeLa cells

HeLa cells were seeded in 6-well or 24 well plates and 6–8 h later were transfected with plasmids using Fugene® 6 according to the manufacturer's instructions (Roche Diagnostics). After 20 h cells were infected with *Salmonella* or processed directly for immunoblotting following solubilization in 150 µl hot 1.5× SDS-PAGE sample buffer.

### RNA-mediated interference

Small interfering RNA (siRNA) SMARTpool (Dharmacon) sequences targeting human Akt1, Akt2, Akt3, PTEN, PDK1, raptor and rictor were diluted and stored according to the manufacturer's instructions. Cells were transfected with siRNA using RNAifect (Qiagen) transfection reagent according to the manufacturer's instructions and infected with *Salmonella* 48 h later. For experiments in which knockdown of p85 was carried out ON-TARGET*plus* SMARTpool siRNA targeting human AKT3, p85α and p85ß was used. HeLa cells were seeded in 6-well plates at a density of 9×10^5^ cells/well and incubated for 16–20 h to yield a monolayer of 50–60% confluency. Cells were transfected using DharmaFect1 reagent (Dharmacon) according to the recommended protocol with 50 nM siRNA for a single target and 25 nM siRNA each for dual targets. Cells were treated with EGF or infected with *Salmonella* 72 h later.

### Imaging of phospho Akt in membrane ruffles

HeLa cells grown on glass coverslips were transfected with plasmid EGFP-mAkt 16–18 hrs prior to infection with *Salmonella*. After 15 min coverslips were fixed in 2.5% paraformaldehyde for 10 min at 37°C, washed in PBS and stained with 1.0 µg/ml WGA-Alexa Fluor 647 (AF647-WGA) for 5 min, washed and fixed for 5 min at 37°C in 2.5% paraformaldehyde. Cells were permeabilized for 5 min with 0.1% saponin, 10% normal goat serum in PBS processed for immunofluorescence using a mouse anti-Akt phospho-serine 473 antibody, followed by AF568-conjugated anti mouse secondary. Coverslips were mounted onto glass slides with Prolong Gold and imaged within 1 week. Z-stacks were acquired for each channel on a Zeiss laser scanning confocal 510 microscope. Laser power and acquisition settings were kept constant for each series of experiments. Cells for imaging were selected on the basis of GFP intensity; only low expressing cells with an average cytoplasmic pixel intensities in the bottom 10% with >1.5−2× background and equivalent GFP intensities between infections were imaged.

### PH domain recruitment to ruffles

HeLa cells on coverslips were transfected with plasmids expressing EGFP-mAkt, EGFP-mAktΔPH, Akt-PH-EGFP, Btk-PH-EGFP, EGFP-TAPP1-PH, PLCδ-PH –EGFP 16–18 hrs prior to infection with *Salmonella*. Cells were fixed at 15 min and processed for immunofluorescence as described above for phospho Akt imaging except that a rabbit anti-LPS antibody, followed by AF568-conjugated rabbit secondary, was used to stain *Salmonella*. Coverslips were mounted in Prolong Gold and imaged within 1 week. Image acquisition and analysis was performed with using a spinning disk confocal microscope [65]. Laser power and acquisition settings were kept constant for each series of experiments. Cells for analysis were selected on the basis of GFP intensity; only low expressing cells with cytoplasmic or nuclear average pixel intensities in the bottom 10% with >1.5−2× background were used. Once a GFP expressing and infected cell was selected, far-red (AF647-WGA), red (AF568), and green (GFP) fluorescence images were acquired sequentially, one set near the bottom of the cell for a representative cytoplasmic section and a second set above the main body to select a section of the protruding ruffle by thresholding on the far-red (AF647-WGA) image. The resulting region-of-interest (ROI) was cloned to the green channel giving the average intensity in the ROI for GFP at the ruffle (GFP_Ruffle_). The average pixel intensity in the green channel at the cytoplasmic section in the cell (GFP_Cyto_) was determined with a circular ROI and the average pixel intensity of the background in the green channel (GFP_Back_) was determined with a circular ROI outside the cell body. The ratiometric calculation was, Recruitment = (GFP_Cyto_−GFP_Back_)/(GFP_Ruffle_−GFP_Back_).

### Microscopy

Confocal images were either captured on a Zeiss LSM510 microscope with 488 nm, 543 nm and 643 nm laser lines or on a spinning disc confocal microscope as previously described [65]. Image analysis and maximum intensity projections were performed with ImageJ v.1.4.1 (written by Wayne Rasband at the U.S. National Institutes of Health and available by anonymous FTP from zippy.nimh.nih.gov) and figures assembled using Adobe Photoshop CS2.

### Statistical Analysis

Unless otherwise noted results are presented as the mean ± S.D. of n = 3 independent experiments. One way Analysis of Variance (ANOVA) combined with the Tukey *post hoc* test was used to determine statistical significance with Prism™ software (GraphPad Software Inc).

## Supporting Information

Figure S1 **Wortmannin is effective at inhibiting EGF-mediated but not** ***Salmonella*** **-mediated Akt phosphorylation.** HeLa cells were pretreated treated with wortmannin (WTM:100 nM) then infected with *Salmonella* for 30 min or 3 hr. For the EGF treated cells agonist was added for 2 min immediately before solubilization at 30 min or 3 hr. Samples were processed for immunoblotting using antibodies to detect phospho Akt (Ser473) and actin.
(TIF)

Figure S2 **Depletion of the class I PI3K regulatory subunits p85α and p85ß does not affect the kinetics of SopB-mediated Akt phosphorylation.** HeLa cells were transfected with siRNAs, specific for p85α and p85ß, for 72 hr then either treated then infected with *Salmonella* WT for 15 min. For time points greater than 15 min monolayers were rinsed to remove non-internalized bacteria and were further incubated in the presence of gentamicin to kill extracellular bacteria. Monolayers were solubilized in sample buffer at the indicated times and processed for immunoblotting using antibodies to detect phospho Akt (Ser473), total Akt or actin.
(TIF)
