# Supplementary figures and images for: Activation of Akt by the Bacterial Inositol Phosphatase, SopB, is Wortmannin Insensitive
Source: PLoS One. 2011 Jul 14;6(7):e22260. doi: 10.1371/journal.pone.0022260 (PMC3136525; doi:10.1371/journal.pone.0022260)

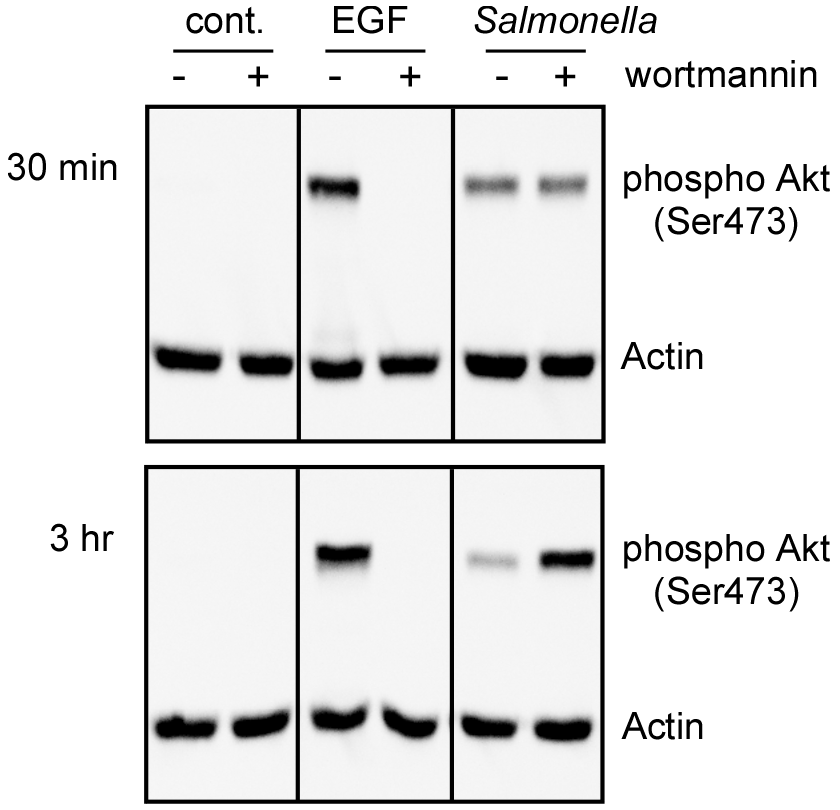

Supplement: Figure S1 — Wortmannin is effective at inhibiting EGF-mediated but not Salmonella -mediated Akt phosphorylation. HeLa cells were pretreated treated with wortmannin (WTM:100 nM) then infected with Salmonella for 30 min or 3 hr. For the EGF treated cells agonist was added for 2 min immediately before solubilization at 30 min or 3 hr. Samples were processed for immunoblotting using antibodies to detect phospho Akt (Ser473) and actin. (TIF) [file pone.0022260.s001.tif]

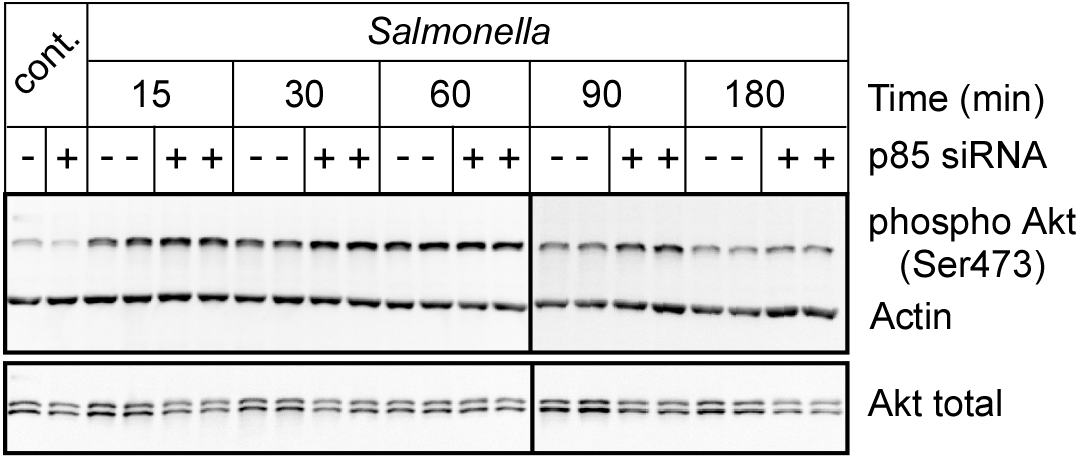

Supplement: Figure S2 — Depletion of the class I PI3K regulatory subunits p85α and p85ß does not affect the kinetics of SopB-mediated Akt phosphorylation. HeLa cells were transfected with siRNAs, specific for p85α and p85ß, for 72 hr then either treated then infected with Salmonella WT for 15 min. For time points greater than 15 min monolayers were rinsed to remove non-internalized bacteria and were further incubated in the presence of gentamicin to kill extracellular bacteria. Monolayers were solubilized in sample buffer at the indicated times and processed for immunoblotting using antibodies to detect phospho Akt (Ser473), total Akt or actin. (TIF) [file pone.0022260.s002.tif]
